# Crystal structure of Zn_2_(HTeO_3_)(AsO_4_)

**DOI:** 10.1107/S2056989021004333

**Published:** 2021-04-27

**Authors:** Felix Eder, Matthias Weil

**Affiliations:** a Institute for Chemical Technologies and Analytics, Division of Structural Chemistry, TU Wien, Getreidemarkt 9/164-SC, A-1060, Vienna, Austria

**Keywords:** crystal structure, oxidotellurate(IV), oxidoarsenate(V), hydrogen bonding, lone pair electrons, stereochemical activity

## Abstract

Single crystals of Zn_2_(HTeO_3_)(AsO_4_), dizinc(II) hydroxidodioxidotellurate(IV) oxidoarsenate(V), were obtained as one of the by-products in a hydro­thermal reaction between Zn(NO_3_)_2_·6H_2_O, TeO_2_, H_3_AsO_4_ and NH_3_ in molar ratios of 2:1:2:10 at 483 K for seven days. The asymmetric unit of Zn_2_(HTeO_3_)(AsO_4_) contains one Te (site symmetry *m*), one As (*m*), one Zn (1), five O (three *m*, two 1) and one H (*m*) site. The Zn^II^ atom exhibits a coordination number of 5 and is coordinated by four oxygen atoms and a hydroxide group, forming a distorted trigonal bipyramid. The hydroxide ion is positioned at a significantly larger distance on one of the axial positions of the bipyramid. The [ZnO_4_OH] polyhedra are connected to each other by corner-sharing to form _∞_^2^[ZnO_3/2_(OH)_1/2_O_1/1_] layers extending parallel to (001). The Te^IV^ atom is coordinated by three oxygen atoms and a hydroxide group in a one-sided manner in the shape of a bis­phenoid, revealing stereochemical activity of its 5*s* ^2^ electron lone pair. The As^V^ atom is coordinated by four oxygen atoms to form the tetra­hedral oxidoarsenate(V) anion. By corner-sharing, [TeO_3_OH] and [AsO_4_] groups link adjacent _∞_^2^[ZnO_3/2_(OH)_1/2_O_1/1_] layers along [001] into a three-dimensional framework structure.

## Abstract 2

The crystal structure of Zn_2_(HTeO_3_)(AsO_4_) consists of _∞_^2^[ZnO_3/2_(OH)_1/2_O_1/1_] layers extending parallel to (001) that are linked by [Te^IV^O_3_OH] and oxidoarsenate(V) groups.

## Chemical context   

Only a few elements have such a diverse crystal chemistry as tellurium, especially in its +IV oxidation state. This can be attributed to the stereochemically active non-bonding 5*s* ^2^ electron pair of Te^IV^ (Galy *et al.*, 1975 ▸) that has a similar space requirement as coordinating ligands and therefore often results in one-sided and low-symmetry coordination spheres around Te^IV^ atoms. An extensive review of the rich crystal chemistry of oxidotellurates(IV) was published recently by Christy *et al.* (2016 ▸).

The peculiar crystal chemistry of Te^IV^ makes it an inter­esting building block in the search for new compounds with crystal structures lacking inversion symmetry. As a prerequisite, a compound must be non-centrosymmetric in order to have ferro-, piezo- or pyroelectric properties or to possess non-linear optical properties (Ok *et al.*, 2006 ▸). Another effect of the electron lone pair and its large space consumption is the frequent formation of open-framework structures in (transition) metal oxidotellurates(IV). Different structure units such as clusters, chains, layers or channels resulting from the presence of oxidotellurate(IV) anions are observed in various crystal structures (Stöger & Weil, 2013 ▸). Introducing secondary anions into transition-metal oxidotellurates(IV) can lead to even more structural diversification. Over the past few years, several anions have been incorporated into metal or transition-metal oxidotellurates, *viz.* sulfates [*e.g*. Cd_4_(SO_4_)(TeO_3_)_3_; Weil & Shirkhanlou, 2017*a* ▸], selenates [*e.g.* Zn_2_(SeO_4_)(TeO_3_); Weil & Shirkhanlou, 2017*b* ▸], carbonates [*e.g.* Pb_5_(SeO_4_)_2_(TeO_4_)(CO_3_); Weil & Shirkhanlou, 2017*c* ▸], nitrates [*e.g.* Ca_6_Te_5_O_15_(NO_3_)_2_; Stöger & Weil, 2013 ▸], phosphates [*e.g.* Co_3_Te_2_O_2_(PO_4_)_2_(OH)_4_; Zimmermann *et al.*, 2011 ▸] or, very recently, arsenates [Cu_5_(TeO_3_)_2_(AsO_4_)_2_; Missen *et al.*, 2020 ▸]. Crystals of Cu_5_(TeO_3_)_2_(AsO_4_)_2_ have been grown by a chemical transport reaction (Binnewies *et al.*, 2012 ▸), starting from CuO, TeO_2_ and As_2_O_5_ at temperatures of 1023 K (source) and 953 K (sink). The title compound, Zn_2_(HTeO_3_)(AsO_4_), however, was obtained at much milder temperatures (483 K) under hydro­thermal conditions.

## Structural commentary   

The asymmetric unit of Zn_2_(HTeO_3_)(AsO_4_) contains one Te, one As, one Zn, one H and five O atoms located either on a special position with site symmetry *m* (Wyckoff position 2 *a*; Te1, As1, O3, O4, O5, H1) or on general positions (Wyckoff position 4 *b*; Zn1, O1, O2). Selected bond lengths are collated in Table 1 ▸.

**Figure 1:**
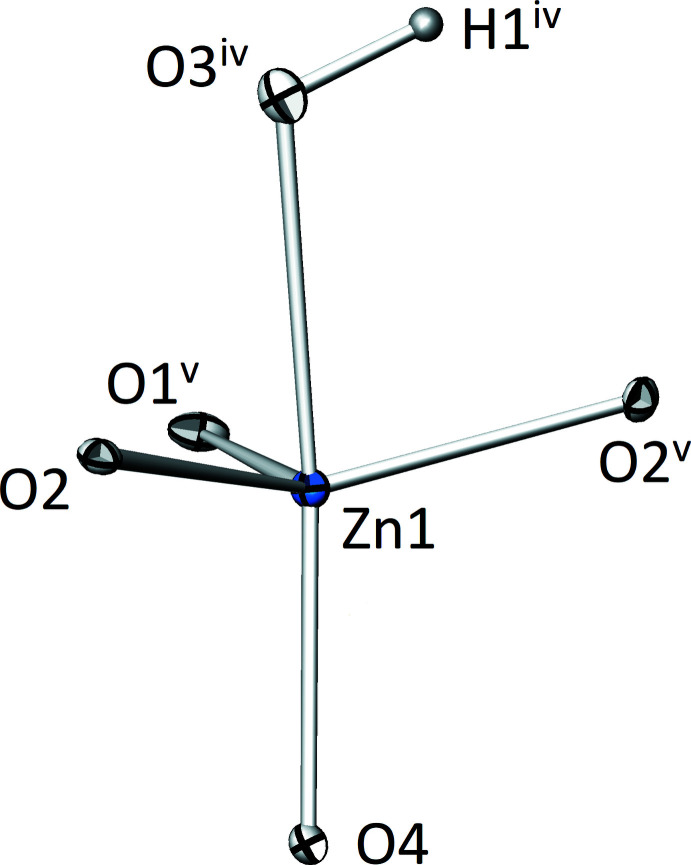
The distorted trigonal–bipyramidal [ZnO_4_OH] polyhedron in the crystal structure of Zn_2_(HTeO_3_)(AsO_4_). Displacement ellipsoids are drawn at the 90% probability level. Symmetry codes refer to Table 1 ▸.

The zinc cation (Zn1) is coordinated by five oxygen atoms with one (O3, as part of the hy­droxy group) being at a significantly longer distance [2.3259 (18) Å] than the other four [1.979 (3)–2.0486 (16) Å]. The resulting polyhedron has the shape of a distorted trigonal bipyramid, with the remote O3 site occupying one of the axial positions and the equatorial positions being slightly tilted towards it (Fig. 1 ▸). The geometry index τ_5_ (Addison *et al.*, 1984 ▸), which is 0 for an ideal square pyramid and 1 for an ideal trigonal bipyramid, amounts to 0.665 for the [ZnO_4_OH] polyhedron. The [ZnO_4_OH] polyhedra are connected to each other by sharing four corners with neighbouring polyhedra to form _∞_^2^[ZnO_3/2_(OH)_1/2_O_1/1_] layers extending parallel to (001). The bond-valence sum (BVS; Brown, 2002 ▸) of Zn1 was calculated to be 1.98 valence units (v.u.) using the values of Brese & O’Keeffe (1991 ▸).

**Figure 2:**
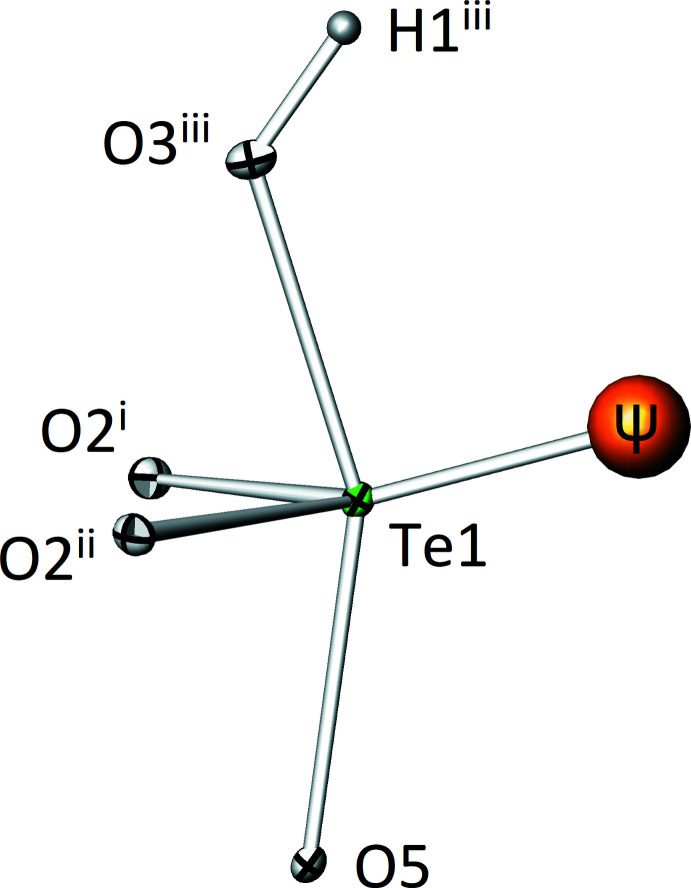
The bis­phenoidal [TeO_3_OH] polyhedron in the crystal structure of Zn_2_(HTeO_3_)(AsO_4_). Displacement ellipsoids are drawn at the 90% probability level. The 5*s* ^2^ electron lone pair (Ψ, orange) is drawn with an arbitrary radius of 0.2 Å. Symmetry codes refer to Table 1 ▸.

The tellurium(IV) atom (Te1) is coordinated by four oxygen atoms with bond lengths in the range 1.880 (2)–2.131 (4) Å. The BVS of Te1 is 4.02 v.u. using the values of Brese & O’Keeffe (1991 ▸) for calculation. With the revised bond-valence values by Mills & Christy (2013 ▸), a lower BVS of 3.86 v.u. was calculated under consideration of the four nearest oxygen atoms. However, the BVS increases to 4.03 v.u. if all oxygen atoms within a distance of up to 3.5 Å are accounted for, as is suggested by Mills & Christy (2013 ▸). The resulting [TeO_3_OH] coordination polyhedron is a bis­phenoid. Under consideration of the space requirement of the 5*s* ^2^ electron lone pair, the corresponding [ΨTeO_3_OH] polyhedron has the shape of a distorted trigonal bipyramid with the non-bonding electron pair occupying an equatorial position (Fig. 2 ▸). The geometry index *τ*_5_ of the [ΨTeO_3_OH] polyhedron is 0.413. The *LPLoc* software (Hamani *et al.*, 2020 ▸) revealed the position of the electron lone pair with resulting fractional coordinates of *x* = 0.7781, *y* = 0, *z* = 0.5519. The radius of the electron lone pair was calculated to be 1.32 Å with a distance of 1.680 Å from the Te1 position. The oxygen atom (O3) of the hy­droxy group is located on an axial position of the [ΨTeO_3_OH] polyhedron. Its hydrogen atom is directed to the O5 site, forming a weak linear hydrogen bond towards the O5 site with a O3⋯O5 distance of 3.213 (5) Å (Table 2 ▸). It is remarkable that the hydrogen atom is located on the oxidotellurate(IV) unit instead of the oxidoarsenate(V) anion given that for 0.1–0.01 *N* solutions, the p*K_b_* value of the [AsO_4_]^3–^ anion is much smaller (2.40 at 291 K) than that of the [TeO_3_]^2–^ anion (6.30 at 298 K) (Weast & Astle, 1982 ▸). Even though the conditions during the hydro­thermal experiment are far from the tabulated values, it is surprising that a difference in the equilibrium constants of almost four orders of magnitude was overridden in the resulting crystal. Nevertheless, as evidenced from a difference-Fourier map and BVS calculations (BVS without contribution of the H atom amounts to 1.15 v.u. for O3), the hydroxide group is located on the oxidotellurate(IV) unit.

The arsenic(V) atom (As1) is coordinated tetra­hedrally by four oxygen atoms with distances in the range 1.673 (2)–1.716 (3) Å. The mean As—O bond length of 1.693 (23) Å is slightly longer than those reported for AsO_4_^3–^ groups [1.667 (18) Å; Schwendtner & Kolitsch, 2019 ▸] or for oxidoarsenate groups in general (also including As—OH bonds, with an overall mean of 1.687 (27) Å; Gagné & Hawthorne, 2018 ▸). The BVS is 4.91 v.u. using the values of Brese & O’Keeffe (1991 ▸) for calculation.

**Figure 3:**
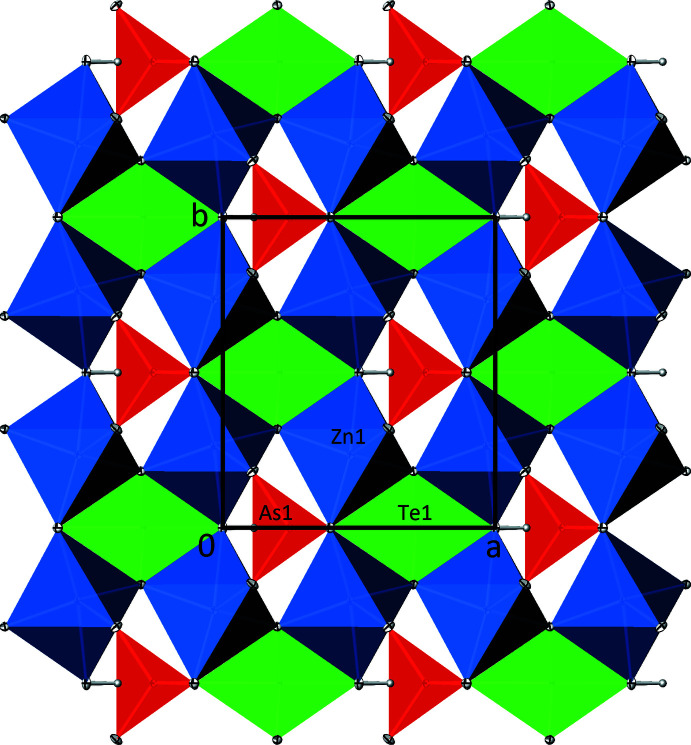
The crystal structure of Zn_2_(HTeO_3_)(AsO_4_) in polyhedral representation, projected onto (001). [ZnO_4_OH] polyhedra are blue, [TeO_3_OH] polyhedra are green and [AsO_4_] tetra­hedra are red; H atoms are represented as grey spheres of arbitrary radius. Displacement ellipsoids are drawn at the 90% probability level.

**Figure 4:**
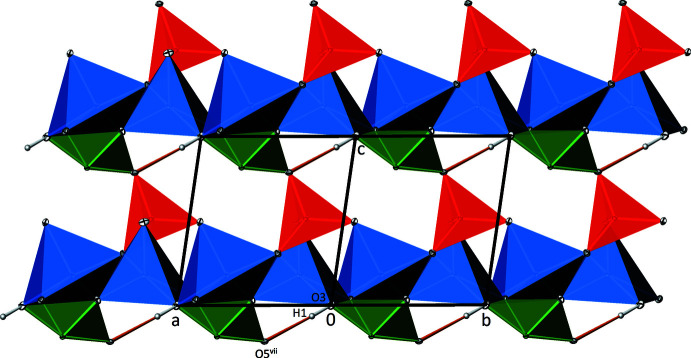
Channels in the crystal structure of Zn_2_(HTeO_3_)(AsO_4_) running parallel to [110]. Colour codes and displacement ellipsoids are as in Fig. 3 ▸. O—H⋯O hydrogen bonds are shown as orange lines.

The crystal structure of Zn_2_(HTeO_3_)(AsO_4_) is built up from _∞_^2^[ZnO_3/2_(OH)_1/2_O_1/1_] layers extending parallel to (001) (Fig. 3 ▸). The [TeO_3_OH] units are situated below a _∞_^2^[ZnO_3/2_(OH)_1/2_O_1/1_] layer and are isolated from each other. An individual [TeO_3_OH] unit shares three corners with two [ZnO_4_OH] polyhedra each, and one corner with an [AsO_4_] tetra­hedron. Likewise, the oxidoarsenate anions, situated above a _∞_^2^[ZnO_3/2_(OH)_1/2_O_1/1_] layer, are isolated from each other, but share corners with other building units: two corners with one [ZnO_4_OH] polyhedron each, one corner with two [ZnO_4_OH] polyhedra and one corner with a [TeO_3_OH] unit. This way, a three-dimensional framework structure is established (Fig. 4 ▸).

**Figure 5:**
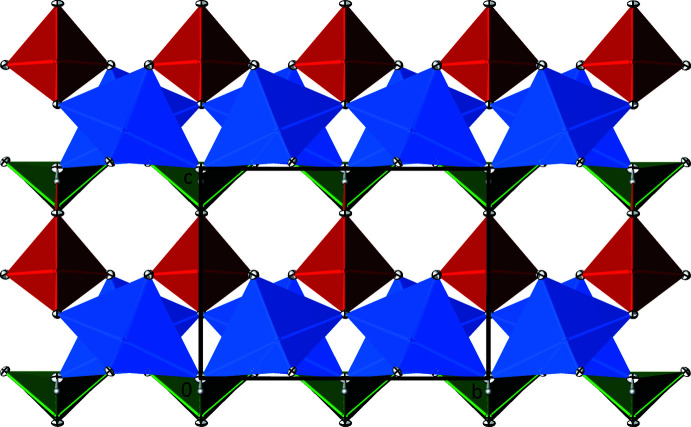
Channels in the structure of Zn_2_(HTeO_3_)(AsO_4_) running parallel to [100]. Colour codes and displacement ellipsoids are as in Fig. 3 ▸.

In the crystal structure, the spatial requirements of the 5*s* ^2^ electron lone pairs at the Te^IV^ atoms lead to the formation of channels parallel to [110] (Fig. 4 ▸). The weak O—H⋯O hydrogen bond is directed across these channels. There are also smaller channels oriented parallel to [100] that, however, remain empty (Fig. 5 ▸).

## Synthesis and crystallization   

Crystals of Zn_2_(HTeO_3_)(AsO_4_) were obtained by hydro­thermal synthesis. The reactants, 0.1949 g of Zn(NO_3_)_2_·6H_2_O (0.670 mmol), 0.0512 g of TeO_2_ (0.321 mmol), 0.1365 g 80%_wt_ of H_3_AsO_4 (aq)_ (0.713 mmol) and 0.22 g of 25%_wt_ NH_3 (aq)_ (3.23 mmol) were weighed into a small Teflon vessel with an inner volume of *ca* 3 ml. The vessel was filled with deionized water to three-quarters of its volume and the reactants were mixed by manual stirring. The Teflon vessel was then put into a steel autoclave and heated to 483 K for 7 d at autogenous pressure. Afterwards, the autoclave was cooled to room temperature within about 4 h. The resulting product was a colourless multi-phase solid. In the X-ray powder pattern of the bulk, Zn_2_(HTeO_3_)(AsO_4_) was found as a by-product, in addition to (NH_4_)Zn(AsO_4_) (Feng *et al.*, 2001 ▸) and the educt TeO_2_ (*α*-TeO_2_; Stehlik & Balak, 1948 ▸). Under a polarizing microscope, small colourless block-shaped crystals of Zn_2_(HTeO_3_)(AsO_4_) were visible that were manually separated for the single-crystal X-ray diffraction study.

## Refinement   

Crystal data, data collection and structure refinement details are summarized in Table 3 ▸. Atom labels and coordinates were standardized with *Structure Tidy* (Gelato & Parthé, 1987 ▸) implemented in *PLATON* (Spek, 2020 ▸). The H atom of the hy­droxy group was located in a difference-Fourier map and was refined freely. The crystal structure was refined under consideration of twinning by inversion, revealing a minor contribution of 3.2 (12)% for the inversion-related twin component.

## Supplementary Material

Crystal structure: contains datablock(s) I. DOI: 10.1107/S2056989021004333/pk2657sup1.cif

Structure factors: contains datablock(s) I. DOI: 10.1107/S2056989021004333/pk2657Isup2.hkl

CCDC reference: 2079463

Additional supporting information:  crystallographic information; 3D view; checkCIF report

## Figures and Tables

**Table 1:** Selected bond lengths (Å)

Te1—O2^i^	1.880 (2)	As1—O5	1.716 (3)
Te1—O2^ii^	1.880 (2)	Zn1—O2^v^	1.979 (3)
Te1—O3^iii^	2.070 (4)	Zn1—O1^v^	1.987 (3)
Te1—O5	2.131 (4)	Zn1—O2	1.993 (3)
As1—O1^iv^	1.673 (2)	Zn1—O4	2.0486 (16)
As1—O1	1.673 (2)	Zn1—O3^vi^	2.3259 (18)
As1—O4	1.709 (3)		

Symmetry codes: (i) x+{\script{1\over 2}}, -y+{\script{1\over 2}}, z+1; (ii) x+{\script{1\over 2}}, y-{\script{1\over 2}}, z+1; (iii) x+1, y, z+1; (iv) x, -y, z; (v) x+{\script{1\over 2}}, -y+{\script{1\over 2}}, z; (vi) x+{\script{1\over 2}}, y+{\script{1\over 2}}, z.

**Table 2:** Hydrogen-bond geometry (Å, °)

*D*—H⋯*A*	*D*—H	H⋯*A*	*D*⋯*A*	*D*—H⋯*A*
O3—H1⋯O5^vii^	0.94 (9)	2.28 (9)	3.213 (5)	179 (7)

Symmetry code: (vii) x, y, z-1.

**Table 3:** Experimental details

Crystal data	
Chemical formula	Zn_2_(HTeO_3_)(AsO_4_)
*M*_r_	446.27
Crystal system, space group	Monoclinic, *C* *m*
Temperature (K)	100
*a*, *b*, *c* (Å)	6.9040 (12), 7.7212 (13), 5.726 (1)
β (°)	101.196 (5)
*V* (Å^3^)	299.43 (9)
*Z*	2
Radiation type	Mo *K*α
μ (mm^−1^)	18.25
Crystal size (mm)	0.06 × 0.04 × 0.03

Data collection	
Diffractometer	Bruker APEXII CCD
Absorption correction	Multi-scan (*SADABS*; Krause *et al.*, 2015 ▸)
*T*_min_, *T*_max_	0.538, 0.748
No. of measured, independent and observed [*I* > 2σ(*I*)] reflections	8261, 1986, 1918
*R*_int_	0.044
(sin θ/λ)_max_ (Å^−1^)	0.915

Refinement	
*R*[*F* ^2^ > 2σ(*F* ^2^)], *wR*(*F* ^2^), *S*	0.019, 0.040, 0.92
No. of reflections	1986
No. of parameters	63
No. of restraints	2
H-atom treatment	All H-atom parameters refined
Δρ_max_, Δρ_min_ (e Å^−3^)	1.79, −1.17
Absolute structure	Refined as an inversion twin
Absolute structure parameter	0.032 (12)

Computer programs: *APEX3* and *SAINT* (Bruker, 2016 ▸), *SHELXT* (Sheldrick, 2015*a* ▸), *SHELXL* (Sheldrick, 2015*b* ▸), *ATOMS for Windows* (Dowty, 2006 ▸) and *publCIF* (Westrip, 2010 ▸).

## supplementary crystallographic information

### Crystal data

**Table:**

Zn_2_(HTeO_3_)(AsO_4_)	*F*(000) = 404
*M**_r_* = 446.27	*D*_x_ = 4.950 Mg m^−^^3^
Monoclinic, *C**m*	Mo *K*α radiation, λ = 0.71073 Å
*a* = 6.9040 (12) Å	Cell parameters from 5391 reflections
*b* = 7.7212 (13) Å	θ = 3.6–40.6°
*c* = 5.726 (1) Å	µ = 18.25 mm^−^^1^
β = 101.196 (5)°	*T* = 100 K
*V* = 299.43 (9) Å^3^	Block, colourless
*Z* = 2	0.06 × 0.04 × 0.03 mm

### Data collection

**Table:**

Bruker APEXII CCD diffractometer	1918 reflections with *I* > 2σ(*I*)
ω– and φ–scans	*R*_int_ = 0.044
Absorption correction: multi-scan (*SADABS*; Krause *et al.*, 2015)	θ_max_ = 40.6°, θ_min_ = 3.6°
*T*_min_ = 0.538, *T*_max_ = 0.748	*h* = −12→12
8261 measured reflections	*k* = −14→14
1986 independent reflections	*l* = −10→10

### Refinement

**Table:**

Refinement on *F*^2^	All H-atom parameters refined
Least-squares matrix: full	*w* = 1/[σ^2^(*F*_o_^2^)] where *P* = (*F*_o_^2^ + 2*F*_c_^2^)/3
*R*[*F*^2^ > 2σ(*F*^2^)] = 0.019	(Δ/σ)_max_ < 0.001
*wR*(*F*^2^) = 0.040	Δρ_max_ = 1.79 e Å^−^^3^
*S* = 0.92	Δρ_min_ = −1.16 e Å^−^^3^
1986 reflections	Extinction correction: SHELXL (Sheldrick, 2015b), Fc^*^=kFc[1+0.001xFc^2^λ^3^/sin(2θ)]^-1/4^
63 parameters	Extinction coefficient: 0.0043 (5)
2 restraints	Absolute structure: Refined as an inversion twin
Hydrogen site location: difference Fourier map	Absolute structure parameter: 0.032 (12)

### Special details

**Table:**

Geometry. All esds (except the esd in the dihedral angle between two l.s. planes) are estimated using the full covariance matrix. The cell esds are taken into account individually in the estimation of esds in distances, angles and torsion angles; correlations between esds in cell parameters are only used when they are defined by crystal symmetry. An approximate (isotropic) treatment of cell esds is used for estimating esds involving l.s. planes.
Refinement. Refined as a two-component inversion twin.

### Fractional atomic coordinates and isotropic or equivalent isotropic displacement parameters (Å^2^)

**Table:**

	*x*	*y*	*z*	*U*_iso_*/*U*_eq_	
Te1	0.70945 (3)	0.000000	0.81776 (3)	0.00418 (6)	
As1	0.24451 (6)	0.000000	0.51174 (7)	0.00440 (9)	
Zn1	0.46889 (7)	0.23359 (4)	0.17917 (8)	0.00521 (7)	
O1	0.1089 (4)	0.1808 (3)	0.4941 (4)	0.0081 (4)	
O2	0.1998 (4)	0.3177 (3)	0.0329 (4)	0.0062 (4)	
O3	−0.0007 (6)	0.000000	0.0002 (6)	0.0087 (6)	
O4	0.3941 (5)	0.000000	0.3064 (6)	0.0063 (5)	
O5	0.3968 (5)	0.000000	0.7868 (6)	0.0065 (5)	
H1	0.114 (13)	0.000000	−0.064 (14)	0.015 (19)*	

### Atomic displacement parameters (Å^2^)

**Table:**

	*U*^11^	*U*^22^	*U*^33^	*U*^12^	*U*^13^	*U*^23^
Te1	0.00465 (12)	0.00392 (11)	0.00381 (11)	0.000	0.00040 (8)	0.000
As1	0.0040 (2)	0.0047 (2)	0.00427 (18)	0.000	0.00031 (16)	0.000
Zn1	0.00389 (15)	0.00549 (13)	0.00595 (15)	0.00021 (15)	0.00023 (11)	0.00043 (13)
O1	0.0090 (10)	0.0078 (10)	0.0068 (9)	0.0039 (8)	−0.0006 (8)	−0.0004 (7)
O2	0.0061 (9)	0.0058 (9)	0.0068 (9)	0.0000 (8)	0.0015 (7)	0.0021 (7)
O3	0.0055 (14)	0.0116 (14)	0.0085 (13)	0.000	0.0001 (11)	0.000
O4	0.0067 (14)	0.0064 (13)	0.0062 (12)	0.000	0.0025 (11)	0.000
O5	0.0049 (13)	0.0103 (14)	0.0041 (11)	0.000	0.0001 (10)	0.000

### Geometric parameters (Å, º)

**Table:**

Te1—O2^i^	1.880 (2)	As1—O5	1.716 (3)
Te1—O2^ii^	1.880 (2)	Zn1—O2^v^	1.979 (3)
Te1—O3^iii^	2.070 (4)	Zn1—O1^v^	1.987 (3)
Te1—O5	2.131 (4)	Zn1—O2	1.993 (3)
As1—O1^iv^	1.673 (2)	Zn1—O4	2.0486 (16)
As1—O1	1.673 (2)	Zn1—O3^vi^	2.3259 (18)
As1—O4	1.709 (3)	O3—H1	0.94 (9)
			
O2^i^—Te1—O2^ii^	96.96 (14)	O2^v^—Zn1—O3^vi^	80.87 (12)
O2^i^—Te1—O3^iii^	79.82 (10)	O1^v^—Zn1—O3^vi^	92.08 (11)
O2^ii^—Te1—O3^iii^	79.82 (10)	O2—Zn1—O3^vi^	71.53 (12)
O2^i^—Te1—O5	83.70 (10)	O4—Zn1—O3^vi^	170.38 (14)
O2^ii^—Te1—O5	83.70 (10)	As1—O1—Zn1^vii^	120.01 (13)
O3^iii^—Te1—O5	155.01 (13)	Te1^viii^—O2—Zn1^vii^	124.12 (13)
O1^iv^—As1—O1	113.14 (19)	Te1^viii^—O2—Zn1	111.74 (13)
O1^iv^—As1—O4	111.36 (10)	Zn1^vii^—O2—Zn1	121.25 (11)
O1—As1—O4	111.36 (10)	Te1^ix^—O3—Zn1^vii^	93.51 (11)
O1^iv^—As1—O5	106.93 (11)	Te1^ix^—O3—Zn1^x^	93.51 (11)
O1—As1—O5	106.93 (11)	Zn1^vii^—O3—Zn1^x^	124.36 (16)
O4—As1—O5	106.69 (17)	Te1^ix^—O3—H1	128 (5)
O2^v^—Zn1—O1^v^	99.25 (11)	Zn1^vii^—O3—H1	109.1 (18)
O2^v^—Zn1—O2	130.47 (12)	Zn1^x^—O3—H1	109.1 (18)
O1^v^—Zn1—O2	121.53 (11)	As1—O4—Zn1	118.20 (8)
O2^v^—Zn1—O4	104.71 (11)	As1—O4—Zn1^iv^	118.20 (8)
O1^v^—Zn1—O4	94.69 (11)	Zn1—O4—Zn1^iv^	123.38 (16)
O2—Zn1—O4	99.06 (12)	As1—O5—Te1	120.43 (18)

Symmetry codes: (i) *x*+1/2, −*y*+1/2, *z*+1; (ii) *x*+1/2, *y*−1/2, *z*+1; (iii) *x*+1, *y*, *z*+1; (iv) *x*, −*y*, *z*; (v) *x*+1/2, −*y*+1/2, *z*; (vi) *x*+1/2, *y*+1/2, *z*; (vii) *x*−1/2, −*y*+1/2, *z*; (viii) *x*−1/2, *y*+1/2, *z*−1; (ix) *x*−1, *y*, *z*−1; (x) *x*−1/2, *y*−1/2, *z*.

### Hydrogen-bond geometry (Å, º)

**Table:**

*D*—H···*A*	*D*—H	H···*A*	*D*···*A*	*D*—H···*A*
O3—H1···O5^xi^	0.94 (9)	2.28 (9)	3.213 (5)	179 (7)

Symmetry code: (xi) *x*, *y*, *z*−1.
